# Perceiving Collision Impacts in Alzheimer’s Disease: The Effect of Retinal Eccentricity on Optic Flow Deficits

**DOI:** 10.3389/fnagi.2015.00218

**Published:** 2015-11-25

**Authors:** Nam-Gyoon Kim

**Affiliations:** Department of Psychology, Keimyung UniversityDaegu, South Korea

**Keywords:** Alzheimer’s disease, retinal eccentricity, tau-dot, optic flow, perceiving collision impacts

## Abstract

The present study explored whether the optic flow deficit in Alzheimer’s disease (AD) reported in the literature transfers to different types of optic flow, in particular, one that specifies collision impacts with upcoming surfaces, with a special focus on the effect of retinal eccentricity. Displays simulated observer movement over a ground plane toward obstacles lying in the observer’s path. Optical expansion was modulated by varying $\dot{\tau }$. The visual field was masked either centrally (peripheral vision) or peripherally (central vision) using masks ranging from 10° to 30° in diameter in steps of 10°. Participants were asked to indicate whether their approach would result in “collision” or “no collision” with the obstacles. Results showed that AD patients’ sensitivity to $\dot{\tau }$ was severely compromised, not only for central vision but also for peripheral vision, compared to age- and education-matched elderly controls. The results demonstrated that AD patients’ optic flow deficit is not limited to radial optic flow but includes also the optical pattern engendered by $\dot{\tau }$. Further deterioration in the capacity to extract $\dot{\tau }$ to determine potential collisions in conjunction with the inability to extract heading information from radial optic flow would exacerbate AD patients’ difficulties in navigation and visuospatial orientation.

## Introduction

Although cognitive impairment characterized by progressive memory loss is the most profound feature of Alzheimer’s disease (AD), vision is also impaired (for reviews, see Cronin-Golomb and Gilmore, 2003; Kirby et al., 2010; Valenti, 2010). One of the less well known forms of visual impairment in AD is optic flow deficit (Page and Duffy, 1999, 2003; Tetewsky and Duffy, 1999; O’Brien et al., 2001; Kavcic and Duffy, 2003; Mapstone et al., 2003, 2008; Monacelli et al., 2003; Duffy et al., 2004; Kavcic et al., 2006; Mapstone and Duffy, 2010). The present study explored whether the optic flow deficit Duffy and colleagues described in AD patients transfers to different types of optic flow, in particular, one that specifies collision impacts with upcoming surfaces, with a special focus on the effect of retinal eccentricity.

### Optic Flow Deficit in AD

**Figure 1:**
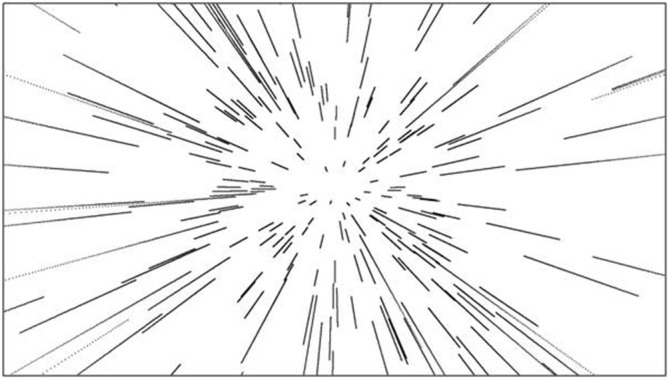
**The radial optic flow produced by observer translation through a 3D cloud of dots.** All dots radiate from the focus of expansion, which corresponds to the observer’s direction of heading.

Optic flow refers to the changing optical structure at a moving point of observation (Gibson, 1966, 1986). Because optic flow is generated by an observer moving in the environment, its structure is specific to the very movement that engendered it. As an observer moves forward along a linear path, her movement engenders a radial flow pattern in which, when characterized as a velocity vector field, optical velocity vectors radiate outward from a point referred to as the *focus of expansion* (FOE; Figure 1). It was Gibson’s contention that this common point from which vectors radiate allows perception of one’s rectilinear direction of movement. Subsequent research has confirmed that human observers use this singularity in the flow field to guide their locomotion (Warren and Hannon, 1988; Royden et al., 1992; for a review, see Warren, 2004).

In a series of studies, Duffy and colleagues investigated AD patients’ perceptual capacity to process optic flow (Page and Duffy, 1999, 2003; Tetewsky and Duffy, 1999; O’Brien et al., 2001; Kavcic and Duffy, 2003; Mapstone et al., 2003, 2008; Monacelli et al., 2003; Duffy et al., 2004; Kavcic et al., 2006; Mapstone and Duffy, 2010). These studies demonstrated that visuospatial disorientation in AD is caused, not only by impairment in landmark orientation mechanisms due to hippocampal damage (Burgess et al., 2006; Laczó et al., 2009), but also by inability to utilize optic flow information. Random-dot cinematograms depicted global optic flow corresponding to an observer’s movement. Displays were comprised of background (or signal) dots that moved radially away from the FOE. Incorporated within this background flow were random (or noise) dots that moved along randomly determined directions in each frame. Participants were asked to identify the location of the FOE, which was deflected to the left or right of the center of the display by a fixed amount. The researchers assessed participants’ ability to process optic flow by varying the level of coherency of the flow (i.e., the ratio of random dots to background dots). Results demonstrated that AD patients performed poorly, exhibiting significantly higher coherent motion thresholds than control participants. However, not all coherent motion induced a similar level of performance in AD patients. When viewing displays in which signal dots moved uniformly along one direction, either vertically or laterally, AD patients performed comparably to control participants with low coherent motion thresholds (Kurylo et al., 1994; Mendola et al., 1995; Rizzo and Nawrot, 1998; Rizzo et al., 2000; O’Brien et al., 2001).

Whereas the overall direction of flow can be easily identified by tracking a single dot from the uniform flow, the FOE can only be identified once the global pattern is extracted from the radial optic flow. To process radial optic flow, the visual system must perform spatio-temporal integration of motion vectors over a large area. Based on their findings demonstrating AD patients’ inability to process radial optic flow, Duffy and colleagues suggested that AD might affect the posterior parietal cortical area, which is known to be involved in human visuospatial capacities (Andersen, 1989, 1997). The area of the posterior parietal cortex that appears to be most prominent in processing optic flow information is the dorsal region of the medial superior temporal area (MSTd). Neurons in the MSTd have been observed to respond selectively to components of optic flow (e.g., expansion/contraction, rotation, translation or a combination of these; Saito et al., 1986; Tanaka and Saito, 1989; Tanaka et al., 1989; Duffy and Wurtz, 1991a,b, 1997, Duffy, 1998) essential for computing heading (i.e., the FOE) from radial optic flow. In fact, the MSTd neurons appear to be able to extract the FOE even when the optic flow undergoes distortion, as when the observer executes pursuit eye movements during locomotion (Page and Duffy, 1999).

Thus, the higher motion coherence threshold reported for radial optic flow in AD patients (O’Brien et al., 2001; Kavcic and Duffy, 2003; Mapstone et al., 2003) may have been due to neural degeneration in posterior parietal cortical areas, particularly in those neurons involved in fronto-parietal interactions. Such degeneration would likely impair integration of the visual cues necessary to compute heading from optic flow (Duffy et al., 2004; Mapstone et al., 2008). Based on similar findings, McKee et al. (2006) suggested that pathology in the visual association area might be the cause of the visual deficits in AD (for a similar argument, see also Rizzo et al., 2000).

### The Magnocellular Deficit Hypothesis in AD

Although neuropathology in the visual association cortices is a prime candidate for visual deficits in AD, another contributing source is defective input from lower-level visual processing areas. MST receives the majority of its inputs from the adjacent middle temporal area (MT, also known as V5). MT has a high concentration of direction-selective neurons devoted to motion processing and constitutes an important station of the dorsal visual pathway along with MST and adjacent areas within the posterior parietal cortex (Zeki, 2004). The dorsal visual pathway receives signals conveyed through the magnocellular subdivisions of the lateral geniculate nucleus (LGN; Maunsell et al., 1990; Merigan and Maunsell, 1993), which, in turn, receives its afferent projection from parasol retinal ganglion cells (M-cells). This subcortical neural stream is referred to as the magnocellular (M) pathway for its projection to the magnocellular layers of the LGN. A separate neural stream projects parallel to the magnocellular pathway from the retina to the primary visual cortex (V1). This pathway originates in midget ganglion cells (or P-cells) and is referred to as the parvocellular (P) pathway for its connection to the parvocellular layers of the LGN. These two pathways are distinct, not only anatomically, but also functionally. Magnocellular cells are more sensitive to higher temporal and lower spatial frequencies and to achromatic contrast, whereas parvocellular cells respond better to lower temporal and higher spatial frequencies and color-opponent signals. Thus, the M-pathway is better suited for processing motion, and the P-pathway is more suited for processing form and color (Livingstone and Hubel, 1987, 1988; Azzopardi et al., 1999; Callaway, 2005).

Abnormal signals originating from the subcortical network of the visual system could compromise higher-level cortical processing. Neuropathological effects of AD on the retina are evidenced as degeneration of retinal ganglion cells and thinning of the retinal nerve fiber layer (Blanks et al., 1996a,b; Paquet et al., 2007). Parasol ganglion cells have been shown to be more susceptible to damage in AD than midget cells (Sadun, 1989; Sadun and Bassi, 1990). Based on this observation, it has been conjectured that a deficit in parasol cells may be responsible for the visual impairments seen in AD, a conjecture referred to as the *magnocellular deficit hypothesis* (Gilmore et al., 2004; Kim and Park, 2010; Kirby et al., 2010; Sartucci et al., 2010; Valenti, 2010). However, subsequent research has failed to confirm Sadun’s finding.

Effects of AD have been also found in subcortical pathways (e.g., Parisi et al., 2001; Kergoat et al., 2002; and for a review, see Kirby et al., 2010). More in line with the magnocellular deficit hypothesis, Gilmore et al. (1994), using a coherent motion paradigm, found that the coherent motion threshold elevated with disease severity. The motion threshold of AD patients correlated with their spatial contrast sensitivity, especially at a high temporal frequency (7.5 Hz; for similar findings, see also Gilmore and Whitehouse, 1995; Gilmore et al., 2004). Other researchers have reported visual deficits in color discrimination, backward masking, and contrast sensitivity at all frequencies in AD patients (Kurylo et al., 1994; Mendola et al., 1995; Adlington et al., 2009). These deficits are thought to be caused by dysfunction in the P pathway, calling into question the magnocellular deficit hypothesis. Nevertheless, the findings of Gilmore et al. (1994, 2004) and Gilmore and Whitehouse (1995) provide evidence that AD disturbs motion processing capacity by degenerating parasol ganglion cells, thereby transmitting flawed signals to the primary visual cortex via the M-pathway.

Visual cortical areas also appear to be vulnerable to AD because of a significant amount of cell loss, particularly in some layers of the primary and secondary visual cortices (V2; Hof and Morrison, 1994; see also Sartucci et al., 2010). Because these cells have long corticocortical projections to MT, such cell loss would disrupt transmission of visual signals from V1 to MT, causing deficits in higher order visual processing. Indeed, AD patients’ elevated motion coherence threshold for radial optic flow (O’Brien et al., 2001; Kavcic and Duffy, 2003; Mapstone et al., 2003) corroborates Hof and Morrison’s contention.

### The Effect of Retinal Eccentricity on Self-Motion Perception

#### Neurophysiological Evidence

As described above, the visual system is comprised of two, largely independent, parallel pathways originating from two different classes of retinal ganglion cells, the midget and the parasol cells. These two classes of cells differ, not only in their structure and function, but also in their number and distribution across the retina. Whereas midget cells account for 80% of retinal ganglion cells, parasol cells comprise only about 10%. Thus, midget cells outnumber parasol cells by a ratio of approximately 8:1. However, ganglion cells are not evenly distributed across the retina. The numbers of both parasol and midget cells peak near the fovea and decline toward the periphery. In the fovea, midget cells contribute about 90% of the ganglion population while parasol cells contribute only 5%. At the periphery, midget cells contribute 40–45%, while parasol cells contribute 20% with the remainder made up of other ganglion cells such as bistratified cells. Thus the ratio of midget cells to parasol cells declines from about 30:1 near the fovea to 3:1 at the periphery (Dacey and Petersen, 1992; Dacey, 1993; see also Azzopardi et al., 1999).

Because of the sharply declining density gradient of midget cells, beyond 6–7° eccentricity, spatial resolution afforded by midget cells falls off drastically from 10 cpd (cycles/degree) to 1 cpd (Dacey, 1993). This severely compromises visual acuity beyond the foveal region. By contrast, the relative abundance of parasol cells in the retinal periphery makes this area more sensitive to fast moving stimuli. Solomon et al. (2002) reported that parasol cells showed an increase in critical fusion frequency (the number of flashes per second at which a flashing light is perceived as being continuous) farther from the fovea.

#### Psychophysical Evidence

Researchers have long attempted to define the processing mode of the visual system based on retinal eccentricity and region of the visual field (Schneider, 1967; Trevarthen, 1968; Held, 1970; Leibowitz and Post, 1982), perhaps in consideration of the uneven distribution, and different density gradients, of retinal ganglion cells and photoreceptors. Central vision within the fovea (~1° eccentricity) and parafovea (~4–5° eccentricity) is thought to be specialized for processing fine details and object identification, whereas peripheral vision (beyond the parafovea) is thought to be sensitive to motion. Recognizing the role that the peripheral retina plays in inducing circular vection or rotary self-motion perception, Dichigans and colleagues (Brandt et al., 1973; Held et al., 1975; Dichigans and Brandt, 1978) postulated a *peripheral dominance hypothesis*. The hypothesis asserts that peripheral stimulation, particularly outside a 30° diameter area of the central visual field, is necessary to elicit self-motion perception.

Gibson (1966, 1986) conceptualized optic flow, particularly that engendered by locomotion along a linear path, as a “melon-shaped family of curves.” This conceptualization suggests that local regions of the optical flow contain different flow patterns. Thus, when gaze is directed along the path of locomotion, a radial flow pattern falls on the central retina, while a lamellar (i.e., parallel) pattern falls on the retinal periphery (for details, see Andersen, 1986). As a result, different regions of the retina are stimulated by different patterns of optical flow. Stoffregen (1985) and Warren and Kurtz (1992) tackled this issue in the contexts of postural control and heading perception, respectively. After finding that the central retina is sensitive to both radial and lamellar flow, and the peripheral retina is sensitive to lamellar, but not radial flow, these researchers postulated the *functional specificity hypothesis*. The hypothesis attributes the functional specialization of retinal location to different regions’ sensitivity to different optical patterns. The researchers attributed the retinal periphery’s apparent dominance for self-motion perception to an experimental confound in which only lamellar flow, the unique flow structure engendered by circular vection, was presented to the retinal periphery.

Crowell and Banks (1993) pointed out that Warren and Kurtz (1992) had confounded retinal location with the structure of flow patterns. By varying flow structures from radial to lamellar and presenting them to various retinal locations, Crowell and Banks showed that the visual system can extract heading information from a variety of flow patterns and retinal locations. These results led Crowell and Banks to put forward the *retinal invariance hypothesis*, which asserts that self-motion perception is determined exclusively by patterns of optical structure, independent of retinal location.

The effect of retinal eccentricity on self-motion perception has been investigated extensively using such perceptual tasks as vection (Brandt et al., 1973; Held et al., 1975; Dichigans and Brandt, 1978; Leibowitz and Post, 1982), postural adjustment (Stoffregen, 1985; Andersen and Dyre, 1989; Bardy et al., 1999), and heading perception (Warren and Kurtz, 1992; Crowell and Banks, 1993; Atchley and Andersen, 1999). Based on the evidence to date, the general consensus is that the consequences of self-motion can be perceived irrespective of retinal eccentricity because both the central and the peripheral retina are sensitive to radial and lamellar flow patterns, a finding consistent with the retinal invariance hypothesis (Bardy et al., 1999).

The radial optic flow deficit in AD (Page and Duffy, 1999, 2003; Tetewsky and Duffy, 1999; O’Brien et al., 2001; Kavcic and Duffy, 2003; Mapstone et al., 2003, 2008; Monacelli et al., 2003; Duffy et al., 2004; Kavcic et al., 2006; Mapstone and Duffy, 2010) is suspected to be caused by an inability to extract a global pattern from optic flow. This may be due to pathological changes in posterior parietal cortical areas, particularly those neurons in the MSTd that respond selectively to various components of radial optic flow. Because MST receives direct input from MT which, in turn, is dominated by signals relayed from the M pathway (Maunsell et al., 1990; Merigan and Maunsell, 1993), defective input originating in the parasol ganglion cells, particularly in the retinal periphery, could aggravate this symptom.

However, the effect of retinal eccentricity on self-motion perception indicates that the central retina is as effective as the peripheral retina in processing optic flow information. This suggests that the observed radial optic flow deficit in AD might be due as much to disturbances in the central retina as to disturbances in the peripheral retina. Although this possibility cannot be ruled out completely, there is reason to suspect that the reported deficit might arise from disturbances in the parasol ganglion cells in the peripheral retina and subsequent neural cells of the streams connecting to those in MT and MST where global optic flow information is processed.

Duffy and colleagues depicted optic flow with the FOE corresponding to the simulated direction of heading deflected from the center of the display by 15–30° either to the left or right from the center (Page and Duffy, 1999, 2003; Tetewsky and Duffy, 1999; O’Brien et al., 2001; Kavcic and Duffy, 2003; Mapstone et al., 2003, 2008; Kavcic et al., 2006). Because their participants were instructed to maintain fixation at the center of the display, the FOE and its radial pattern of the optic flow was projected to the peripheral retina. Thus, it is reasonable to suspect that the poor performance of AD patients could have been due to abnormalities in the parasol ganglion cells in the retinal periphery and the neural structures in the subsequent relay stations of the subcortical and cortical pathways, including those in MT and MST. This interpretation (the magnocellular deficit hypothesis discussed earlier) is consistent with research findings that selective degeneration of the neural structures comprising the magnocellular pathway leads to motion perception impairments in AD (Sadun, 1989; Sadun and Bassi, 1990; Gilmore et al., 1994, 2004; Gilmore and Whitehouse, 1995; Blanks et al., 1996a,b; Parisi et al., 2001; Kergoat et al., 2002; Paquet et al., 2007; Sartucci et al., 2010).

Although it is reasonable to suspect neuropathology in the magnocellular and the dorsal networks of the visual system as the cause of the radial optic flow deficit in AD, the data provided by Duffy and colleagues are insufficient to confirm the retinal eccentricity effect of the deficit. Knowing which way one is heading is certainly important for navigating successfully through the environment. The heading information that facilitates locomotion is the FOE contained in the radial optic flow. However, an observer executes various movements to get about in a stable environment, for example, “beginning forward locomotion, ceasing locomotion, reversing locomotion; steering toward a specific place or object; approaching without collision; avoiding obstacles; pursuit of a moving object; and avoiding a moving object” (Gibson, 2009, p. 264). Each of these movements produces a unique pattern of optic flow. Even forward locomotion gives rise to different optic flow patterns, depending on how the path curves (Warren et al., 1991; Kim and Turvey, 1998; Kim et al., 2000).

Thus, two questions arise: (1) Is the optic flow deficit observed in AD patients limited to the radial optic flow engendered by locomotion along a linear path, or is the deficit a more general symptom encompassing other types of optic flow? (2) If the capacity to process optic flow information is impaired in AD, does this impairment arise from selective dysfunction in the parasol ganglion cells in the retinal periphery and their projection to the magnocellular pathway or from dysfunction of all retinal neurons?

### The Present Study

The present study was conducted to address these questions. A task used previously (Kim, 2013) was employed in the present study. To assess the effect of retinal eccentricity on the perception of self-motion, Kim depicted graphically what (Gibson (2009), p. 265) termed “approaching without collision.” For Gibson, this action can be accomplished “by so moving as to cancel the centrifugal flow of the optic array at the moment when the contour of the object or the texture of the surface reaches that angular magnification at which contact is made”. Gibson’s v “formula” was formalized mathematically by Lee (1976). Put simply, as an observer approaches an object, the optical solid angle subtended by the object expands. Lee proved that the inverse of the relative rate of optical expansion specifies the time to contact (TTC) between the observer and the object, assuming that approach velocity is held constant. Lee referred to this optical variable as τ. Further research has demonstrated that this optical variable is used in the control of a variety of activities (for a review, see Lee, 2009).

Lee (1976) also demonstrated that the time derivative of τ ($\dot{\tau }$ or “tau-dot”) can be used to control the impact of collision with an upcoming surface. Specifically, when $\dot{\tau }$ ≥ −0.5, the corresponding optical states specify that the impending collision will be soft (i.e., deceleration is sufficient so that the actor would stop before or at the obstacle). When $\dot{\tau }$ < −0.5, the corresponding optical states specify that the impending collision will be hard (i.e., the actor would collide with the obstacle). Thus, animals can approach an obstacle without collision by keeping $\dot{\tau }$ near a cut-off value of −0.5. Subsequent research confirmed that human observers are not only sensitive to this optical variable (Kim et al., 1993; Andersen et al., 1999), but also use it in the visual control of braking (Yilmaz and Warren, 1995; Rock and Harris, 2006).

Note that $\dot{\tau }$ is the time derivative of τ, whereas τ is defined as the inverse of the relative rate of optical expansion. Thus, sensitivity to $\dot{\tau }$ would require that the visual system respond selectively to optical looming. Although research on neural substrates for detecting heading is too numerous to list here, research on neural mechanisms for time-to-contact estimation, especially in humans, is scarce. One study using human functional magnetic resonance imaging (fMRI; Field and Wann, 2005) demonstrated that looming patterns specifying an imminent collision activate the dorsal pathway (particularly areas MT and MST, which are known to be sensitive to optic flow). It is well documented that MSTd neurons encode the components of optic flow, of which expansion is one (Saito et al., 1986; Tanaka et al., 1989; Tanaka and Saito, 1989; Duffy and Wurtz, 1991a,b, 1997; Duffy, 1998). Because looming is characterized as the expansion of an object’s image in the image plane, Browning (2012) proposed a template model of MSTd cells that can estimate TTC concurrently with heading directly from optic flow. Dysfunction of neurons in areas MT and MST would compromise, not only heading estimation, but also TTC and $\dot{\tau }$ estimation.

Kim (2013) assessed the effect of masking either the center or the periphery of the visual field on young adults’ perception of collision impact. When the central field was masked, participants responded consistently (in conformity to the tau-dot hypothesis). When the peripheral field was masked, participants’ performance was inconsistent (violating the tau-dot hypothesis). In the current study, I used the same collision impact perception task to elucidate the optic flow deficit in AD. Peripheral vision facilitates the perception of collision impact, even with no central stimulation. If AD alters signals feeding the magnocellular pathway, this perceptual capacity will be severely compromised in AD patients exposed to the peripheral vision condition. Because healthy young adults performed this task erratically when the peripheral field was masked, it was expected that AD patients would perform as poorly as control participants in the central vision condition.

Kim’s (2013) Experiment 1 was largely replicated in the present study. Displays simulated observer movement toward three red octagonal road signs in the middle of the roadway parallel to the ground plane. Simulation was engendered under the constraint that the expansion of the scene resulting from forward translation maintained a fixed value of $\dot{\tau }$ throughout the approach. The effect of retinal eccentricity was assessed by masking displays either centrally (peripheral vision) or peripherally (central vision). Mask size ranged from 10° to 30° in diameter in steps of 10°.

## Materials and Methods

### Participants

Twenty-three AD patients (9 males and 14 females) and 23 healthy elderly control (EC) participants (14 males and 9 females) participated in the study. AD patients were recruited from a local university hospital in Daegu (mean age = 70.4 years, *SD* = 6.1 years; mean education = 8.1 years, *SD* = 4.7 years). Selection of AD patients was based on the diagnostic guidelines of the National Institute of Neurological and Communicative Disorders and Stroke-Alzheimer’s Disease and Related Disorders Association (NINCDS-ADRDA) for probable or possible AD (McKhann et al., 1984). Additional evaluations included a neurological examination and either CT or MRI scan to exclude other causes of dementia. Dementia severity was assessed by the Korean adaptation (Kwon and Park, 1989) of the Mini Mental State Examination (MMSE; Folstein et al., 1975) and the Clinical Dementia Rating (CDR) Scale (Morris, 1993). For the AD patients, the mean MMSE score was 19.7 (*SD* = 3.5); and all had a CDR score of 0.5 or 1 (mean *CDR* = 0.98, *SD* = 0.10). EC were comprised of temporary workers in the University Maintenance Department (mean age = 67.6 years, *SD* = 4.9; mean education = 9.7 years, *SD* = 3.3 years; mean *MMSE* = 27.8, *SD* = 2.1. AD and EC groups were matched for age, *t*_(44)_ = −1.73, *p* > 0.05, and years of education, *t*_(44)_ = 1.31, *p* > 0.05. Demographic data for the participants are presented in Table 1.

**Table 1:** **Demographic characteristics of participants**.

	AD	Elderly controls	*p*-Values
Age	70.4 ± 6.1	67.6 ± 4.9	*p* > 0.05
Education	8.1 ± 4.7	9.7 ± 3.3	*p* > 0.05
MMSE	19.7 ± 3.5	27.8 ± 2.1	*p* < 0.0001
N (M, F)	23 (14,9)	23 (9,14)

All participants had normal or corrected-to-normal vision and reported no history of ophthalmologic disorder. Participants received a nominal ($5) fee for their participation in the experiment. Two AD patients and one control participant selected only one response out of two choices throughout the experiment. Their data were excluded from analysis.

### Ethics Statement

The study was approved by a local research ethics committee. After complete description of the study to the participants, written informed consent was obtained in accordance with the Declaration of Helsinki.

### Apparatus

The experiment was conducted in a laboratory at the hospital for the AD patients and in one of the Psychology Department laboratories for the EC group. Displays were presented on a 42-inch and a 32-inch monitor, respectively, for the AD and the EC group, with a pixel resolution of 1280 H × 1024 V and frame rate of 60 Hz. The AD group viewed the display binocularly at a distance of approximately 60 cm; and the EC group viewed the display at a distance of approximately 45 cm, subtending a field of view of 76.6° H × 47.7° V and 75.8° H × 47.4° V, respectively. No physical constraints on head movement were imposed during the experiment.

### Stimuli

**Figure 2:**
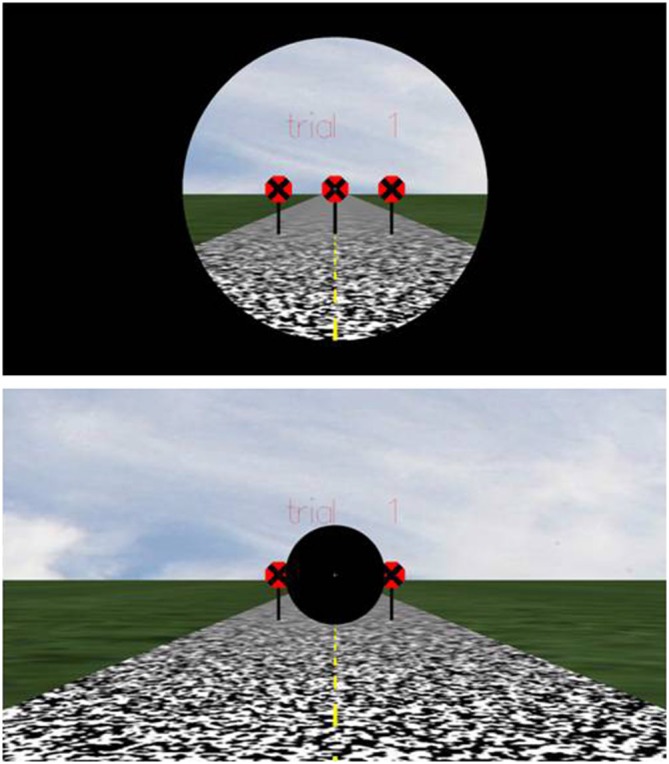
**Displays used in the study: (top) central vision condition (mask *size* = 30°); (bottom) peripheral vision condition (mask *size* = 10°)**.

The simulated scene showed a green pasture-textured ground plane under a partially cloudy sky. The ground plane was 200 m wide and 400 m deep and 1.6 m below eye level. A straight section of 8 m wide roadway rendered by a random check texture was shown in perspective (Figure 2). Three octagonal red road signs with black crosses and a radius of 0.5 m were located at the midpoint of the visible roadway. Each road sign was attached to the ground plane by a 0.1 m × 1.6 m black rectangular bar. All signs were equidistant from the observation point, but each was 2 m from the next. The observer’s path was directed toward the middle road sign, and the center of the road sign coincided with the observation point.

The displays were engendered under the constraint that $\dot{\tau }$ remained constant throughout the approach. The displays terminated (i.e., went blank) when the observation point reached 1.6 m before the target.

Displays were masked either centrally or peripherally. In the central vision condition, an aperture at the center of the display blocked the scene corresponding to the peripheral visual field from view, but left the central visual field visible (top panel of Figure 2). In the peripheral vision condition, a black disk in the center of the display obscured the central visual field from view, but left the peripheral field visible (bottom panel of Figure 2).

### Design

Two variables ($\dot{\tau }$ and mask size) were controlled in the experiment. $\dot{\tau }$ varied from −0.08 to −0.92 in steps of 0.12. Approach was initiated with an initial velocity of 13.20 m/s at a distance of 20.12 m from the target. This combination yielded displays lasting from 1.5 s for the short event ($\dot{\tau }$ = −0.92) to 3.5 s for the longest event ($\dot{\tau }$ = −0.08). Mask size diameter varied over three levels: 10°, 20°, and 30°. These manipulations yielded a 2 (vision type: central vs. peripheral) × 8 ($\dot{\tau }$) × 3 (mask size) design with one repetition for a total 48 completely randomized trials. Vision type was controlled between subjects, and $\dot{\tau }$ and mask size were controlled within subjects.

### Procedure

Within each group, half the participants were presented with the central vision condition and the other half were presented with the peripheral vision condition. Prior to the experiment, the experimenter collected MMSE and CDR ratings from AD patients but only MMSE scores from EC.

Because of their age, level of education, and lack of familiarity with this type of experiment, the experimenter controlled the computer, the application that controlled the stimulus presentation, and recorded their responses.

Trials were initiated when the experimenter pressed the space bar to trigger the display. Participants were told to watch the display while maintaining visual fixation on the marker located in the center of the screen throughout the trial. Upon termination of the display, participants were asked to indicate whether their approach would result in collision or no collision with the signs, judging from the level of deceleration to that point.

To familiarize participants with the task, the application was demonstrated using two $\dot{\tau }$ values (−0.15 and −0.25) that resulted in no collisions and two $\dot{\tau }$ values (−0.85 and −0.95) that resulted in collisions. Participants then were given a 6-trial practice session prior to the experiment to allow them to become familiar with the experimental setup. Six values of $\dot{\tau }$ (−0.08, −0.11, −0.14, −0.96, −0.99, −1.02) were combined with initial distance of 28.35 m and initial velocity of 19.14 m/s to produce the six practice trials. Mask sizes of 25.0° and 2.5° were used for the central and peripheral vision conditions, respectively. Feedback was provided during the practice trials but not during the experiment.

### Data Analysis

Responses were coded as 0 for a no collision event and 1 for a collision event. Half of the trials resulted in no collisions ($\dot{\tau }$ values of −0.08, −0.20, −0.32, and −0.44) and the other half in collisions ($\dot{\tau }$ values of −0.56, −0.68, −0.80, and −0.92). Thus, when responses are combined across the $\dot{\tau }$ values in each condition of mask size, the averaged mean should not differ from 0.50 according to the $\dot{\tau }$ hypothesis. One sample *t*-test was performed using averaged mean responses in each condition of mask size in each vision type to assess the effect of vision type (i.e., retinal eccentricity). A more detailed analysis was conducted with a mixed design analysis of variance (ANOVA) with vision type, $\dot{\tau }$ and mask size as independent variables. In addition, performance (i.e., response accuracy) of the two groups was assessed by recoding responses according to the $\dot{\tau }$ hypothesis (if response is “no collision” when $\dot{\tau }$ ≥ −0.5 or “collision” when $\dot{\tau }$ < −0.5, it was recoded as “correct”; otherwise it was recoded as “incorrect”).

## Results

### EC

**Figure 3:**
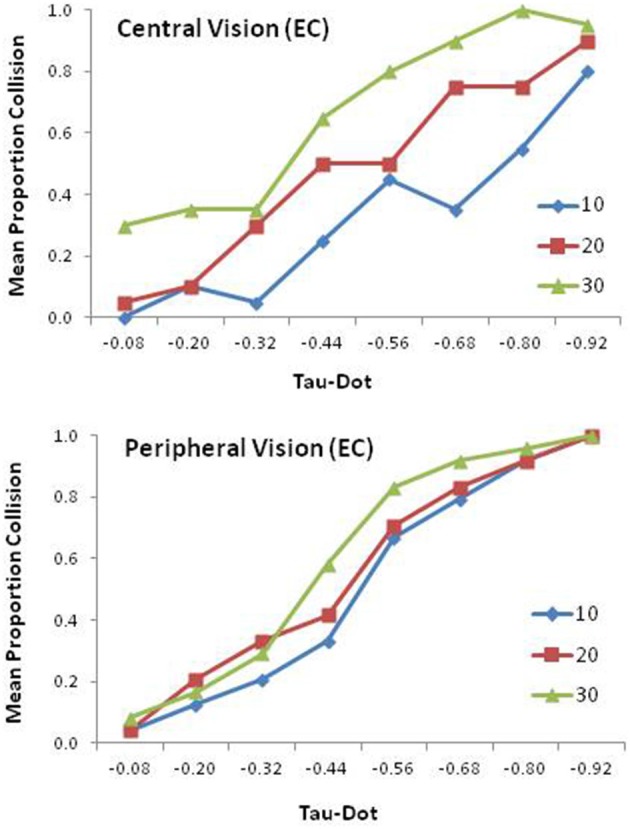
**Mean proportion of collision responses for elderly controls as a function of tau-dot and mask size in the central (top panel) and peripheral (bottom panel) vision conditions**.

Mean proportion of collision judgments is presented as a function of $\dot{\tau }$ for each condition of mask size in Figure 3 for central (top panel) and peripheral (bottom panel) vision. In the central vision condition, the averaged means were 0.32 (*SD* = 0.13), 0.48 (0.15), and 0.66 (0.18) for mask sizes of 10°, 20°, and 30°, respectively. In the peripheral vision condition, the averaged means for the same mask sizes were 0.51 (*SD* =* 0*.10), 0.56 (0.07), and 0.61 (0.13), respectively. The means of the latter two conditions (20° and 30°) were significantly different from 0.50 [*t*_(11)_ = 2.73, *p* < 0.05; *t*_(11)_ = 2.72, *p* < 0.05].

An ANOVA indicated significant main effects of $\dot{\tau }$, *F*_(7,140)_ = 94.73, *p* < 0.0001, $\eta_{p}^{2}$ = 0.83, and mask size, *F*_(2,40)_ = 16.88, *p* < 0.0001, $\eta_{p}^{2}$ = 0.46, and a marginally significant effect of vision type, *F*_(1,20)_ = 4.07, *p* = 0.57, $\eta_{p}^{2}$ = 0.17. Vision type interacted with mask size, *F*_(2,40)_ = 5.52, *p* < 0.01, $\eta_{p}^{2}$ = 0.22. A simple effects analysis indicated significant effects of mask size in the central vision condition, *F*_(2,19)_ 12.10, *p* < 0.0001, $\eta_{p}^{2}$ = 0.56, and of vision type in the 10° mask size condition, *F*_(1,20)_ = 1.89, *p* < 0.01, $\eta_{p}^{2}$ = 0.44.

Vision type also interacted with $\dot{\tau }$ at a marginally significant level, *F*_(7,140)_ = 2.04, *p* = 0.054, $\eta_{p}^{2}$ = 0.09. A simple effects analysis indicated a significant effect of $\dot{\tau }$ for both conditions of vision type [*F*_(7,14)_ = 23.32, *p* < 0.0001, $\eta_{p}^{2}$ = 0.92, for central vision; *F*_(7,14)_ = 47.59, *p* < 0.0001, $\eta_{p}^{2}$ = 0.96, for peripheral vision]. The effect of vision type was significant at $\dot{\tau }$ = −0.68, *F*_(1,20)_ = 4.76, *p* < 0.05, $\eta_{p}^{2}$ = 0.19; $\dot{\tau }$ = −0.80, *F*_(1,20)_ = 5.46, *p* < 0.05, $\eta_{p}^{2}$ = 0.21; and $\dot{\tau }$ = −0.92, *F*_(1,20)_ = 6.60, *p* < 0.05, $\eta_{p}^{2}$ = 0.25.

### AD Patients

**Figure 4:**
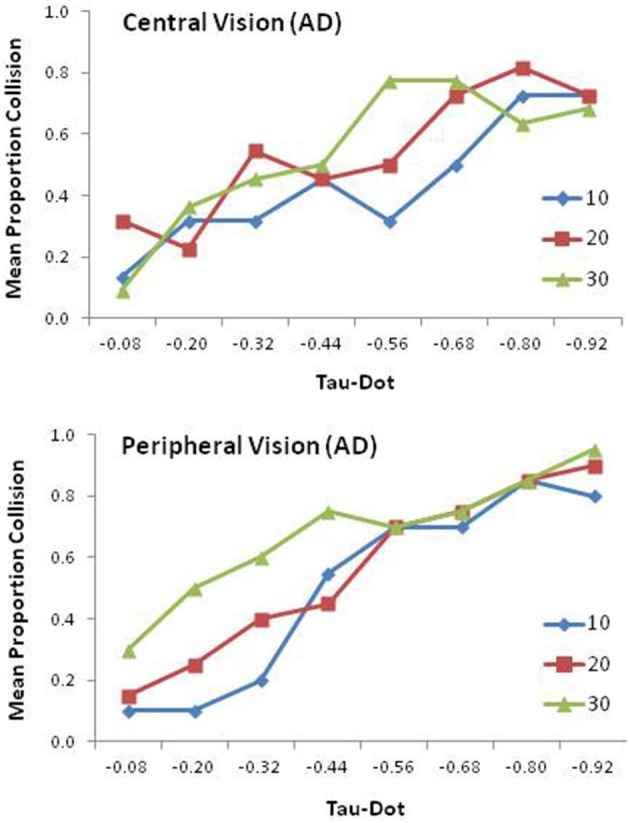
**Mean proportion of collision responses for AD patients as a function of tau-dot and mask size in the central (top panel) and peripheral (bottom panel) vision conditions**.

In Figure 4, the mean proportion of collision judgments is presented as a function of $\dot{\tau }$ for each condition of mask size for central (top panel) and peripheral (bottom panel) vision. The averaged means for the three mask sizes (10°, 20°, and 30°) in the central vision condition were 0.44 (*SD* = 0.28), 0.54 (0.20), and 0.53 (0.23), respectively. The corresponding means in the peripheral vision condition were 0.50 (*SD* = 0.19), 0.56 (0.15), and 0.68 (0.26), respectively. However, none of these 6 means differed from 0.50.

The results of an ANOVA with vision type, $\dot{\tau }$ and mask size as variables revealed only a significant main effect of $\dot{\tau }$, *F*_(7,133)_ = 23.63, *p* < 0.0001, $\eta_{p}^{2}$ = 0.55.

### EC vs. AD Patients

Mean proportion accuracies for EC were 0.74 (*SD* = 0.09) and 0.82 (0.07), respectively, for central and peripheral vision. For AD patients, the corresponding accuracies were 0.65 (*SD* = 0.15) and 0.71 (0.07) for central and peripheral vision, respectively. A 2 (group) by 2 (vision type) ANOVA confirmed main effects of group, *F*_(1,39)_ = 9.88, *p* < 0.01, $\eta_{p}^{2}$ = 0.20, and vision type, *F*_(1,39)_ = 5.28, *p* < 0.05, $\eta_{p}^{2}$ = 0.12. For reference, the corresponding means for young adults reported by Kim (2013) were 0.80 and 0.84, respectively, for central and peripheral vision.

## Discussion

The current study addressed two questions. First, is the optic flow deficit in AD reported by Duffy and colleagues confined to radial optic flow or is it a more general symptom reflecting the inability of AD patients to perceive the consequences of their own movement, irrespective of movement type? Second, do abnormalities in the parasol ganglion cells, particularly those in the retinal periphery, and the neural structures in the subsequent relay stations of the subcortical and cortical pathways, including those in MT and MST, contribute to the optic flow deficit in AD?

With respect to the first question, in this study what Gibson (2009) referred to as “approaching without collision” was employed as an instance of self-motion. A graphic simulation was used to compare AD patients’ sensitivity to $\dot{\tau }$, an optical variable that specifies collision impacts with upcoming surfaces, with that of age- and education-matched healthy elderly controls. In previous studies directed at sensitivity to $\dot{\tau }$, the responses of young adult participants matched the pattern typically observed in a categorical perception study. That is, judgments tended to be “no collision” when $\dot{\tau }$ ≥ −0.5, or “collision” when $\dot{\tau }$ < −0.5, largely conforming to the $\dot{\tau }$ hypothesis, but tended to be at chance level near −0.5 (Kim et al., 1993).

To address the second question, following Kim (2013), displays were masked either centrally or peripherally. In the central vision condition, an aperture in the center of the display blocked from the observer’s view the scene corresponding to the peripheral visual field, but left the central visual field visible (top panel of Figure 2). In the peripheral vision condition, a black disk in the center of the display blocked the central visual field from view, but left the peripheral field visible (bottom panel of Figure 2).

The effect of vision type was dramatic for EC. As shown in Figure 3, the three mask size graphs were quite distinct in the central vision condition (top panel) but collapsed onto each other in the peripheral condition (bottom panel). Significantly, the response patterns from the two vision type conditions were similar to those reported by Kim (2013) for young adults. Specifically, for central vision the small aperture condition (10°) elicited more “no collision” judgments, whereas the large aperture condition (30°) elicited more “collision” judgments, with the means of these two conditions differing significantly from 0.50 [*t*_(9)_ = −4.40, *p* < 0.01; *t*_(9)_ = 2.88, *p* < 0.05], replicating the pattern observed in Kim (2013) (i.e., biased responses toward “no collision” at smaller apertures but toward “collision” at larger apertures). This finding was corroborated by an ANOVA revealing a significant interaction between vision type and mask size. This interaction was caused by elderly controls responding differently under the different mask sizes in the central vision condition, but not in the peripheral vision condition.

The response patterns of AD patients stand in stark contrast with those of EC. AD patients’ sensitivity to $\dot{\tau }$ became largely indiscernible in the central vision condition (top panel of Figure 4) and in the peripheral vision condition (bottom panel of Figure 4). Moreover, the effects of vision type and mask size were negligible, exerting little influence on AD patients’ perception of collision impacts.

The findings of the present study can be summarized as follows. First, for EC, response accuracy was slightly degraded, particularly in the central vision condition (0.72 vs. 0.80), compared with that of the young adults reported in Kim (2013) possibly due to aging. However, aging appears to have little influence on sensitivity to $\dot{\tau }$, with response patterns for both central and peripheral vision largely replicating those of young adults of the Kim study. In particular, peripheral vision, which facilitated the perception of collision impacts for young adults, still played a facilitating role in EC, irrespective of mask size (bottom panel of Figure 3), which produced comparable response accuracies (0.80 vs. 0.84). Interestingly, the central vision condition elicited similar biased responses to those observed in young adults (i.e., favoring “no collision” at smaller apertures but “collision” at larger apertures). This tendency was corroborated by an ANOVA that revealed a significant main effect of mask size (top panel of Figure 3), which also was observed for young adults in Kim.

AD patients did not exhibit the characteristic pattern observed in the $\dot{\tau }$ studies (Kim et al., 1993; Kim, 2013). Peripheral vision appeared to lose its efficacy in facilitating $\dot{\tau }$ perception. More significantly, AD patients’ performance degradation was not limited to peripheral vision. Central vision elicited inconsistent responses in $\dot{\tau }$ perception for both the young adults as in Kim and the elderly controls in the present study. However, central vision was quite reliable, eliciting consistent patterns of bias under different aperture conditions. Not only did overall performance of AD patients decline in the central vision condition (0.65), but the effect of mask size was no longer statistically significant (top panel of Figure 4).

Taken together, the present results demonstrate that AD patients’ optic flow deficit is not limited to radial optic flow but extends as well to the optical pattern engendered by $\dot{\tau }$ and the capacity to perceive impending collision impacts. Pathological changes in the posterior parietal cortical areas are suspected to cause the optic flow deficits (Hof and Morrison, 1994; Duffy et al., 2004; McKee et al., 2006; Mapstone et al., 2008). Degeneration of neurons in MSTd that respond selectively to optic flow components such as expansion/contraction, rotation, translation, or a combination of these (Saito et al., 1986; Tanaka et al., 1989; Tanaka and Saito, 1989; Duffy and Wurtz, 1991a,b, 1997; Duffy, 1998) would certainly disable the capacity to perform the spatio-temporal integration needed to extract a global pattern, such as the FOE or $\dot{\tau }$, from optic flow. The preceding visual area MT projecting directly to MST is primarily driven by magnocellular input. The magnocellular pathway with its primary receptors in the retina extending to the primary visual cortex relays visual information related to motion. Numerous research findings have shown that this subcortical pathway is particularly susceptible to AD. Based on this, the magnocellular deficit hypothesis (Gilmore et al., 2004; Kirby et al., 2010; Sartucci et al., 2010; Valenti, 2010) conjectures that defective signals from the neural structures that comprise the magnocellular pathway (particularly the parasol ganglion cells in the retinal periphery) contribute to the visual deficits seen in AD.

Selectively masking central or peripheral areas of the visual field and presenting the optic flow pattern engendered by $\dot{\tau }$ either to the central or the peripheral field was intended to reveal optic flow deficits in AD as a function of retinal eccentricity. Degraded performance of AD patients in the peripheral vision condition corroborated the conjecture, but degraded performance in the central vision condition did not. These conflicting results provide inconclusive evidence for selective damage in the subcortical structures, particularly the mangnocellular pathway, as the source of visual dysfunction in AD.

MST in the posterior parietal cortex has been identified as the locus of optic flow processing. Interestingly, MST via MT is thought to be primarily driven by magnocellular input. If so, performance decline in the peripheral vision condition by AD patients is predictable, given its structural damage due to AD despite intact signals conveyed through the magnocellular stream. In the central vision condition, the peripheral retina was excluded from stimulation by a mask, keeping the magnocellular stream virtually devoid of visual signals. Under this condition, performance declined in AD patients. Nevertheless, their level of performance (0.65) exceeded chance (0.50, i.e., 1 out of 2). With the magnocellular network blocked, no signals should have reached MST. Nevertheless, AD patients were able to partition optical patterns engendered by $\dot{\tau }$ into two distinct states, albeit not as reliably as in the peripheral vision condition, but still beyond the chance level. The relatively accurate level of performance demonstrated by the EC group (0.74) or young adults (0.80) in Kim (2013) in the central vision condition is even more remarkable.

It is not clear how the visual information needed for computing $\dot{\tau }$ to determine collision impacts, particularly in the central vision condition, reached MST in either the present study (both EC and AD patients) or the Kim (2013) study. The suggestion that MT is primarily driven by magnocellular input was based on the finding that selective inactivation of the magnocellular pathway at the level of LGN eliminated responses of most neurons in MT. Parvocellular inactivation, on the other hand, produced far less effect on responses in MT (Maunsell et al., 1990). Nevertheless, Maunsell et al. characterized the effect (albeit minor) as unequivocal evidence of parvocellular contribution to MT responses.

Interestingly, Huk et al. (2002) conducted a series of fMRI experiments within the human MT+ complex in an effort to identify functionally distinct subregions corresponding to MT and MST identified in primate studies. Based on their results, the researchers divided this region into two distinct subregions with one (putatively MT) exhibiting retinotopic organization but failing to respond to peripheral (>10° from the vertical meridian) stimulation; whereas the other (putatively MST) showing the opposite pattern. In an experiment designed to assess the cortical representation of the central (a central disk of 4° radius) and peripheral (a peripheral annulus of 4° inner radius and 16.5° outer radius) parts of the visual field, the researchers presented (either expanding or contracting) radial optic flow patterns to each of these two portions of the visual field alternately. In this experiment, the researchers found that the putative MST responded to the central stimulation in five of the eight participants.

It appears, then, that stimulation of the central visual field modulates some activity in MST neurons. Although it is not clear the passage of the signals that activated MST responses in the Huk et al. (2002) study, perhaps, the same signals may account for the performance observed in the central vision condition of the present study as well as that of the Kim (2013) study.

Duffy and colleagues (Page and Duffy, 1999, 2003; Tetewsky and Duffy, 1999; O’Brien et al., 2001; Kavcic and Duffy, 2003; Mapstone et al., 2003, 2008; Monacelli et al., 2003; Duffy et al., 2004; Kavcic et al., 2006; Mapstone and Duffy, 2010) demonstrated that the inability to extract heading information from radial optic flow contributes to AD patients’ difficulties in navigation and visuospatial orientation. Additional impairment in extracting $\dot{\tau }$ from looming optical patterns would exacerbate AD patients’ navigational difficulties. Indeed, Uc et al. (2006) reported that drivers with AD are prone to respond unsafely in collision avoidance situations, thus increasing their chances for a rear-end collision. Based on the results of tests assessing the visual and cognitive capabilities of AD patients, the authors identified several factors as predictors for unsafe outcomes by drivers with AD. However, decreased sensitivity to $\dot{\tau }$ may be a more likely contributing factor to AD drivers’ propensity to respond ineffectively in collision avoidance situations. Additional research is needed to confirm this conjecture.

## Conclusion

The present results demonstrate that AD patients’ sensitivity to $\dot{\tau }$ is severely compromised, impairing their ability to perceive collision impacts with upcoming surfaces. These results extend Duffy and colleagues’ findings that AD patients’ optic flow deficits are not limited to radial optic flow but also to the optical pattern engendered by $\dot{\tau }$. AD’s detrimental effects on the capacity to process $\dot{\tau }$ in conjunction with the impaired capacity to extract heading information from radial optic flow would severely compromise AD patients’ capacity to navigate through a cluttered environment.

## Conflict of Interest Statement

The author declares that the research was conducted in the absence of any commercial or financial relationships that could be construed as a potential conflict of interest.
